# Inhibitory Effects of Raw-Extract *Centella asiatica* (RECA) on Acetylcholinesterase, Inflammations, and Oxidative Stress Activities via In Vitro and In Vivo

**DOI:** 10.3390/molecules25040892

**Published:** 2020-02-17

**Authors:** Zetty Zulikha Hafiz, Muhammad ‘Afif Mohd Amin, Richard Muhammad Johari James, Lay Kek Teh, Mohd Zaki Salleh, Mohd Ilham Adenan

**Affiliations:** 1 Faculty of Applied Sciences, Universiti Teknologi MARA, 40450 Shah Alam, Selangor Darul Ehsan, Malaysia; zulikha87@gmail.com; 2 Atta-ur-Rahman Institute for Natural Product Discovery (AuRIns), Level 9, FF3 Puncak Alam Campus, Universiti Teknologi MARA, 42300 Puncak Alam, Selangor Darul Ehsan, Malaysia; 3 Integrative Pharmacogenomics Institute (iPROMISE), Universiti Teknologi MARA Selangor Branch, Puncak Alam Campus, 42300 Puncak Alam, Selangor Darul Ehsan, Malaysiarichard@puncakalam.uitm.edu.my (R.M.J.J.); tehlaykek@puncakalam.uitm.edu.my (L.K.T.); zakisalleh@puncakalam.uitm.edu.my (M.Z.S.); 4 Faculty of Pharmacy, Universiti Teknologi MARA Selangor Branch, Puncak Alam Campus, 42300 Puncak Alam, Selangor Darul Ehsan, Malaysia; 5 Universiti Teknologi MARA Pahang Branch, 26400 Bandar Tun Abdul Razak, Jengka, Pahang Darul Makmur, Malaysia

**Keywords:** *Centella asiatica*, anti-acetylcholinesterase, anti-inflammatory, antioxidant, lipopolysaccharides, in vitro, in vivo

## Abstract

*Centella asiatica* (*C. asiatica*) is one of the medicinal plants that has been reported to exert comprehensive neuroprotection in vitro and in vivo. In view of this, the present study was performed to investigate the effect of ethanolic extract of *C. asiatica*, designated as raw-extract of *C. asiatica* (RECA) in reducing the acetylcholinesterase (AChE), inflammations, and oxidative stress activities via both in vitro (SH-SY5Y and RAW 264.7 cells) and in vivo (Sprague Dawley rats). Quantitative high-performance liquid chromatography analysis reveals that RECA contains a significantly high proportion of glycosides than the aglycones with madecassoside as the highest component, followed by asiaticoside. Treatment of SH-SY5Y cells with RECA significantly reduced the AChE activity in a concentration-dependent manner with an IC_50_ value of 31.09 ± 10.07 µg/mL. Furthermore, the anti-inflammatory and antioxidant effects of RECA were evaluated by lipopolysaccharides (LPS)-stimulated RAW 264.7 cells. Our results elucidated that treatment with RECA significantly suppressed the level of pro-inflammatory cytokine/mediators and oxidative stress released in a concentration-dependent manner. Interestingly, these patterns of inhibition were consistent as observed in the LPS-induced neuroinflammation Sprague Dawley rats’ model. The highest concentration used in the two models presented the most significant results. Herein, our findings strongly suggest that RECA may offer therapeutic potential for the treatment of Alzheimer’s disease through inhibiting the AChE, inflammation, and oxidative stress activities.

## 1. Introduction

Alzheimer’s disease (AD), which is associated with progressive memory loss and impairment in cognitive and behavioral functions, is the most common neurodegenerative disease worldwide [1]. The etiopathogenesis of this disease is multifactorial and recent pieces of evidence have demonstrated that the deficits in central cholinergic neurotransmission in the brain, inflammatory injury, and induction of oxidative stress are also interrelated in the onset and progression of AD [2,3,4]. Suppressing the acetylcholinesterase activity in the brain is one of the most popular targets for AD treatment to ameliorate cognitive ability. This enzyme, when in excess, could further lead to depletion of the acetylcholine concentration that will eventually result in deficit neurotransmission within the synaptic region [5]. Consequently, most of the prescription drugs to manage the symptoms and halt the progression of AD are working as acetylcholinesterase inhibitors such as rivastigmine, donepezil, galantamine, and memantine [6,7].

Lipopolysaccharides (LPS), a pro-inflammatory bacterial mimetic derived from the outer membrane of gram-negative bacteria, is often used in the research field to promote neuroinflammation in vitro or in vivo. Once a model is stimulated by LPS, the levels of pro-inflammatory cytokines/mediators and oxidative stress would become significantly elevated. Prolonged elevation of this imbalanced inflammation and oxidative stress would eventually accelerate the manifestation and progression of AD [2,4,8]. Therefore, inhibition of the inflammatory response is crucial in restricting the development of this disease.

Currently, there is no highly effective medicine that can prevent, halt, or reverse the progressive course of AD [9,10]. Although there are well-established (semi-)synthetic drugs currently used for the management of AD, most of them have several adverse effects. Thus, public and scientific attention has turned toward phytopharmaceuticals as promising candidates for drug development owing to their wide therapeutic potential, superior safety, and consumer acceptability [11,12]. For instance, evodiamine (Evo), an extract of *E. rutaecarpa* Bentham, is widely used in traditional Chinese medicine as herbal medicine as it is believed to exhibit anti-AD, anti-inflammatory, antinociceptive, and thermoregulatory activities [13,14,15]. Meanwhile, a herbal medicine called ninjin’yoeito (NYT), a form of a traditional Japanese medicine (Kampo), has demonstrated both cognitive improvement and a reduction of AD-related depression to the patients that received a combination of donepezil supplemented with NYT [16].

*C. asiatica (L.) Urban*, or locally known as “pegaga” in Malaysia, is a psychoactive medicinal plant belonging to the *Apiaceae* family (formerly *Umbelliferae*) [17]. It is a small perennial plant that is commonly eaten raw as a salad (ulam) and used as a topical product, especially among the older generation in Malaysia [18]. Various uses are claimed for the plant, which has long been recognized by Ayurvedic and Chinese medical practitioners particularly for its rejuvenator and cognitive enhancer effects [19,20,21]. Moreover, it also has been shown to possess neurological, antioxidant, anti-inflammatory, and anticancer activities; yet, its mechanism of action is not clearly portrayed. The most prominent group of active compounds identified from this plant is pentacyclic triterpenoid saponins including asiaticoside, madecassoside, and their sapogenins (madecassic acid and asiatic acid), which are believed attributable to its wide spectrum of therapeutic actions [22,23]. A study done by Won et al. [24] reported that madecassic acid isolated from *C. asiatica* more potently suppressed the inflammatory mediators than madecassoside did via the downregulation of NF-kB activation in RAW 264.7 macrophage cells. This may point to the different affinities exhibited by madecassoside and madecassic acid for cellular membranes. Moreover, results from Park et al. [25] demonstrated that titrated *C. asiatica* (TECA), which comprises 29–30% madecassic acid, effectively inhibited LPS-induced inflammatory responses. However, the triterpene components in *C. asiatica* are not always the same due to the geographical origin, diverse environmental conditions, and different accessions [26]. The absence reports of adverse effects in any clinical studies further corroborate in the classification of this plant as a Class 1 herb (one that can safely be consumed when used appropriately) in the Botanical Safety Handbook [27].

Based on these rationales, this study is mainly devoted to finely evaluate the effect of ethanolic *C. asiatica* extract designated as raw-extract *C. asiatica* (RECA) in inhibiting the acetylcholinesterase (AChE) activity and suppressing inflammation and oxidative stress levels in vitro and in vivo. Results of this study will provide extra insights for using *C. asiatica* as an alternative for the treatment of AD.

## 2. Results

### 2.1. Quantitative High-Performance Liquid Chromatography (HPLC) Analysis

**Figure 1:**
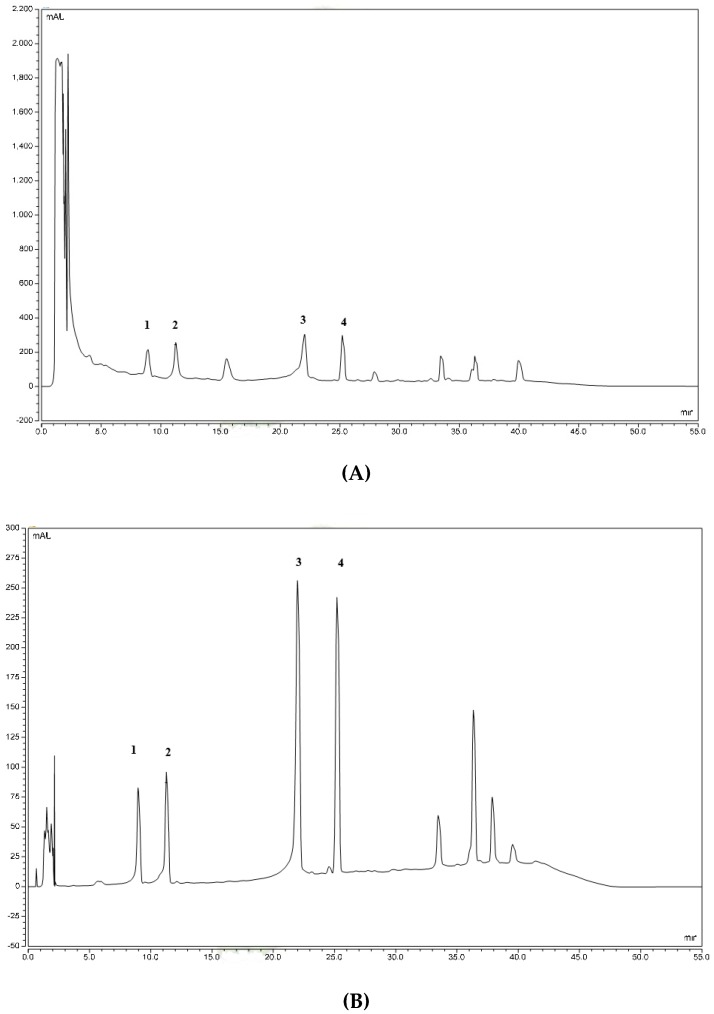
Chromatograms of raw-extract of *C. asiatica* (RECA) (**A**) and standards mixture (**B**). (**1**): madecassoside; (**2**): asiaticoside; (**3**): madecassic acid; (**4**): asiatic acid.

Figure 1A,B shows a chromatogram of RECA and the reference standards which are asiaticoside, madecassoside, asiatic acid, and madecassic acid. Four triterpenes in RECA, which are madecassoside, asiaticoside, madecassic acid, and asiatic acid were quantified by using the HPLC gradient method with an ultraviolet (UV) detector at 200 nm (Table 1). This extract contains a high proportion of glycosides than the aglycones, in which madecassoside is the highest component. Several researchers were able to detect these triterpenes within the wavelength range of 200–220 using a gradient system comprising water and acetonitrile [28,29,30].

### 2.2. Effect of RECA on Viability of SH-SY5Y and RAW 264.7 Cells

**Figure 2:**
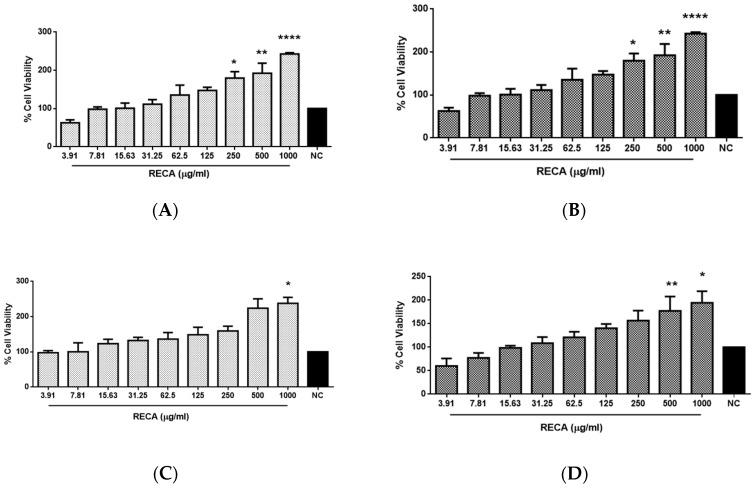
Cytotoxicity effect of RECA against SH-SY5Y (**A**,**B**) and RAW 264.7 (**C**,**D**) cell lines after 24 h (**A**,**C**) and 48 h (**B**,**D**) of exposure using MTT assay at the concentration ranging from 3.91 to 1000 μg/mL. NC = negative control (untreated cells). Results are expressed as the mean ± SEM in triplicate. * *p* ≤ 0.05, ** *p* ≤ 0.01, and **** *p* ≤ 0.0001 compared to NC denoted as 100%.

The cytotoxic effect of RECA at a concentration ranging from 3.91 to 1000 μg/mL was investigated on neuroblastoma cells (SH-SY5Y) and murine microglia cells (RAW 264.7) by using MTT assay. The effect of RECA on SH-SY5Y and RAW 264.7 cell lines was evaluated after 24 h and 48 h (Figure 2) of cell exposure to the extract. The cell viability percentage was calculated by measuring the resulting intracellular purple formazan formed from the reduction of MTT solution by the presence of mitochondrial dehydrogenase in viable cells. It was observed that cell viability on both cells increased with the increase in concentrations of RECA. However, the level of cell viability was slightly lowered at 48 h as compared to 24 h of exposure. RECA at the highest concentration of 1000 μg/mL showed maximal cell viability at both hours of exposure (* *p* ≤ 0.05 and **** *p* ≤ 0.0001). No significant degree of cytotoxicity was observed according to the modified US National Cancer Institute and Geran et al. [31] criteria, as follows: IC_50_ ≤ 20 µg/mL = highly cytotoxic; IC_50_: 21–200 µg/mL = moderately cytotoxic; IC_50_: 201–500 µg/mL = weakly cytotoxic; and IC_50_ > 501 µg/mL = non-cytotoxic. Since the concentration of RECA up to 1000 μg/mL did not exhibit cytotoxicity against both cell lines, these concentrations were selected for subsequent experiments.

### 2.3. Inhibitory Effect of RECA on AChE Activity

**Figure 3:**
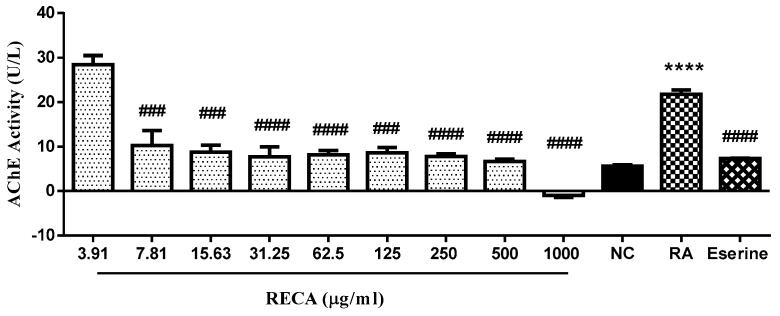
Effect of RECA on acetylcholinesterase activity after 24 h of exposure at the concentration ranging from 3.91 to 1000 μg/mL. NC = negative control (untreated cells) and RA = differentiated SH-SY5Y cells. Results are expressed as the mean ± SEM in triplicate. **** *p* ≤ 0.0001 compared NC, whereas ^###^ *p* ≤ 0.01 and ^####^ *p* ≤ 0.0001 as compared to RA.

To evaluate the inhibitory effect of RECA on AChE activity in vitro, SH-SY5Y cells were used in this study. The differentiated cells were exposed to RECA at a concentration ranging from 3.91 to 1000 μg/mL for 24 h. The enzyme activity was estimated by Ellman’s method and expressed where unit of enzyme catalyzes the production of 1 µmole of thiocholine per minute under the assay conditions (pH 7.5 and room temperature). Eserine at 0.16 µM was used as the standard AChE inhibitor in this study. Based on Figure 3, differentiated SH-SY5Y cells (RA) showed a significant increase (**** *p* ≤ 0.0001) in AChE activity as compared to undifferentiated cells. However, AChE activity in differentiated cells showed a significant decrease in a concentration-dependent manner when the cells were treated with RECA for 24 h. Interestingly, RECA at the highest concentration (1000 μg/mL) showed significant maximal inhibition (^####^ *p* ≤ 0.0001) on AChE activity as compared to RA alone. The IC_50_ values for RECA and eserine were found at values of 31.09 ± 10.07 μg/mL and 0.242 ± 0.227 μg/mL, respectively.

### 2.4. Inhibitory Effect of RECA on Anti-Inflammatory Activities

**Figure 4:**
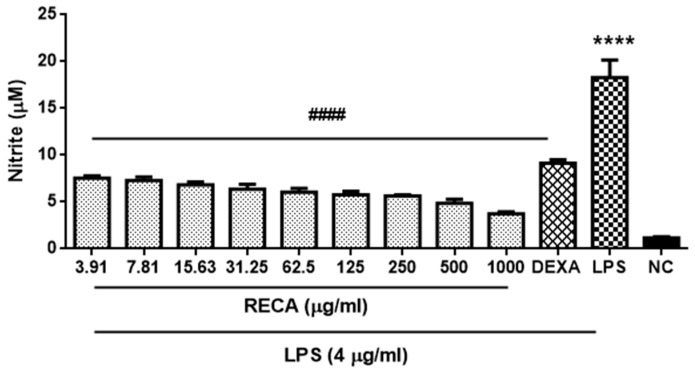
Effect of RECA on nitrite production. Result is expressed as the mean ± SEM in triplicate. NC = negative control (untreated cells), DEXA = dexamethasone and LPS = lipopolysaccharides. **** *p* ≤ 0.0001 compared to NC, whereas, ^####^ *p* ≤ 0.0001 compared to LPS.

In order to elucidate the capability of RECA on suppressing the pro-inflammatory cytokines and mediators, initially, we examined the effect of RECA on inhibiting the nitrite level in LPS-induced RAW 264.7 cell line. The nitrite level in the cellular supernatants was assessed using Griess reagent. Dexamethasone at 5 µM was used as the reference drug. Nitrite (<2 µM) was present in unstimulated RAW 264.7 microglia cells; however, the level of nitrite was markedly increased (**** *p* ≤ 0.0001) after exposure to LPS for 24 h. Treatment with RECA at various concentrations ranging from 3.91 up to 1000 µg/mL significantly decreased (^####^ *p* ≤ 0.0001) the subsequent LPS-induced nitrite production in a concentration-dependent manner with the maximal inhibitory effect seen at a dosage of 1000 µg/mL. The present study also found the nitrite-suppressing effect of treatment with RECA at the highest dosage (1000 µg/mL) to be better than the reference drug, dexamethasone (Figure 4). RECA was found to exhibit the greatest maximal 50% inhibitory effect of nitrite with a value of 5.205 ± 3.13 μg/mL.

**Figure 5:**
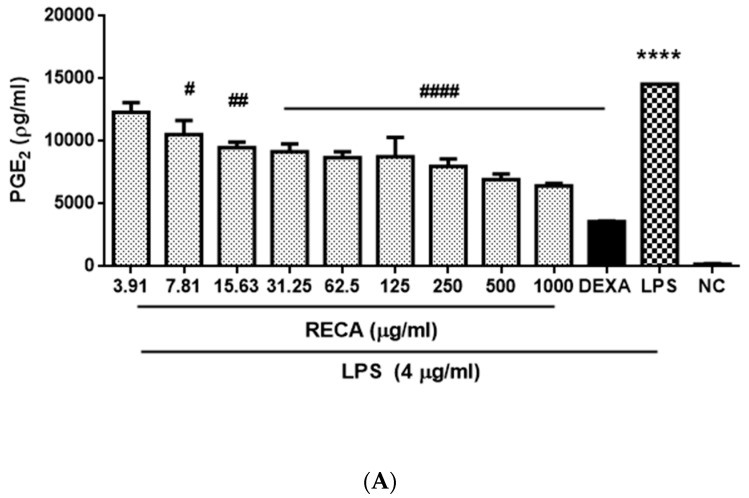

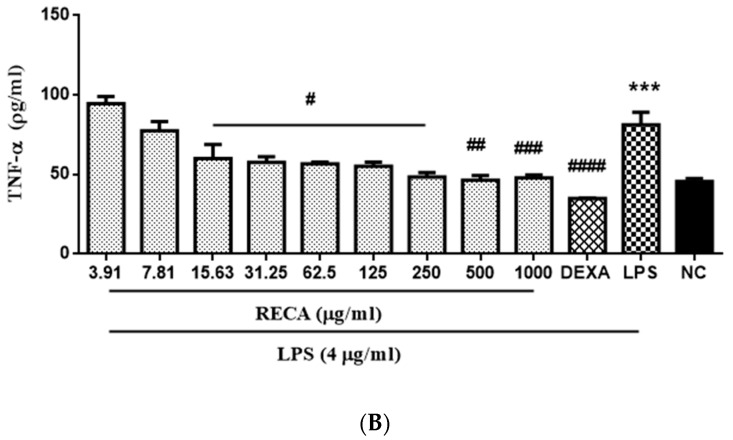
Effect of RECA on (**A**) PGE_2_ and (**B**) TNF-α expressions. NC = negative control (untreated cells), DEXA = dexamethasone, and LPS = lipopolysaccharides. Results are expressed as the mean ± SEM in triplicate. *** *p* ≤ 0.001 and **** *p* ≤ 0.0001 compared to NC, whereas, ^#^ *p* ≤ 0.05, ^##^ *p* ≤ 0.01, ^###^ *p* ≤ 0.001, and ^####^ *p* ≤ 0.0001 compared to LPS.

As RECA showed the greatest inhibition activity of nitrite production, we also examined its suppressive effect toward other pro-inflammatory cytokine/mediators, which are prostaglandin E₂ (PGE_2_) and tumor necrosis factor alpha (TNF-α). Dexamethasone at 5 µM and 6 µM were used as the reference drug for PGE_2_ and TNF-α analyses, respectively. RAW 264.7 macrophage cells that were stimulated with 4 µg/mL LPS markedly (*** *p* ≤ 0.001 and **** *p* ≤ 0.0001) showed an increment in PGE_2_ and TNF-α expressions as compared to that generated by control. The result in Figure 5 showed a similar inhibition effect as demonstrated in Figure 4 as RECA inhibited the expression of PGE_2_ and TNF-α in the macrophages stimulated with LPS in a concentration-dependent manner. Moreover, RECA at the highest concentration (1000 µg/mL) showed a remarkable inhibition effect (*** *p* ≤ 0.001) as compared to untreated cells in both assays. RECA was found to exhibit a maximal 50% inhibitory effect of PGE_2_ and TNF-α with values of 261.4 ± 14.2 µg/mL and 744 ± 14.38 µg/mL, respectively.

### 2.5. Inhibitory Effect of RECA on Antioxidant Activities

**Figure 6:**
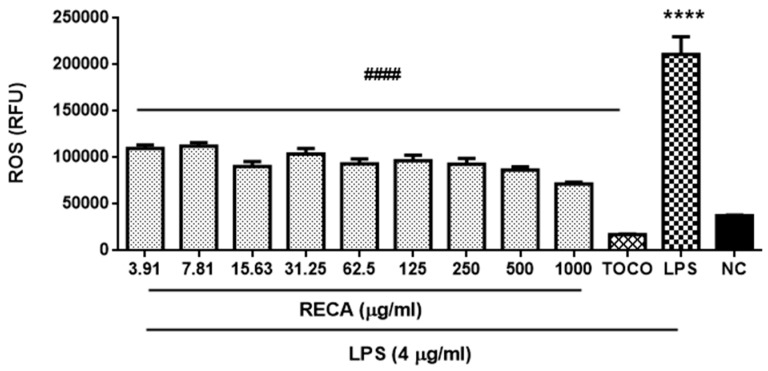
Effect of RECA on reactive oxygen species (ROS) production. NC = negative control (untreated cells), TOCO = α-tocopherol and LPS = lipopolysaccharides. Results are expressed as the mean ± SEM in triplicate. **** *p* ≤ 0.0001 compared to untreated cells (NC), whereas, ^####^ *p* ≤ 0.0001 compared to LPS.

Having observed the encouraging anti-inflammatory effects of RECA, we further explore its potential antioxidant properties. To this end, we first examined the effect of RECA on reactive oxygen species (ROS) production in RAW 264.7 macrophages stimulated with LPS. The ROS production was quantified using the stable nonpolar dye 2′,7′-dichlorodihydrofluorescein diacetate (H_2_DCF-DA), which readily diffuses into the cells and is enzymatically hydrolyzed by intracellular esterase to non-fluorescent 2′,7′-dichlorofluorescein (DCFH), which is rapidly oxidized to highly fluorescent DCF in the presence of ROS. α-tocopherol at 160 µM was used as the reference drug. A minimal ROS level (<40,000 RFU) was present in unstimulated cells; however, a noteworthy generation of ROS (**** *p* ≤ 0.0001) was observed once cells were stimulated with LPS (Figure 6). RECA was observed inhibiting the production of ROS in a concentration-dependent manner with RECA yielding the greatest suppression (^#####^ *p* ≤ 0.0001) at the highest concentration (1000 µg/mL). RECA was found to exhibit greatest the maximal 50% inhibitory effect of ROS with a value of 20.15 ± 2.54 μg/mL.

**Figure 7:**
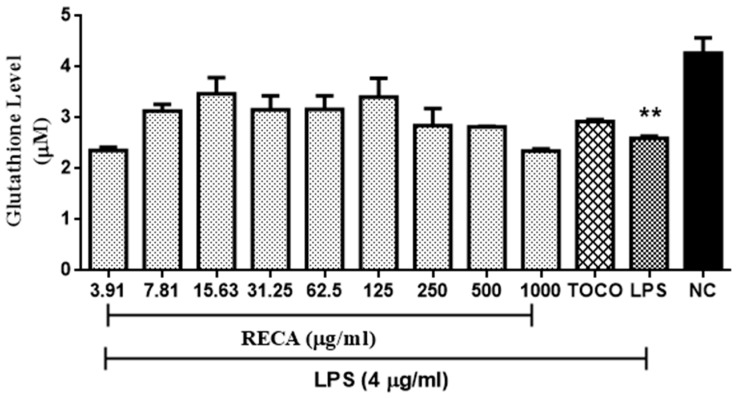
Effect of RECA on glutathione (GSH) content. NC = negative control (untreated cells), TOCO = α-tocopherol, and LPS = lipopolysaccharides. Results are expressed as the mean ± SEM in triplicate. ** *p* ≤ 0.01 as compared to NC.

Reduced glutathione (GSH) content in LPS-induced RAW 264.7 cell line was also quantified, as shown in Figure 7. α-tocopherol at 250 µM was used as the reference drug for this assay. Unstimulated RAW 264.7 microglia cells were found to exhibit a high level of GSH (≤1.46 µM); however, the GSH content was significantly (** *p* ≤ 0.00) depleted following stimulation with LPS. In general, incubation of RECA on stimulated cells prevented the LPS-induced depletion in GSH level in a dose-dependent manner up to the concentration of RECA at 125 ug/mL. Nonetheless, further incubation of RECA at a concentration of 250 µg/mL to 1000 µg/mL elicited suppression effect on the GSH content.

### 2.6. Inhibitory Effect of RECA on AChE Activity In Vivo

**Figure 8:**
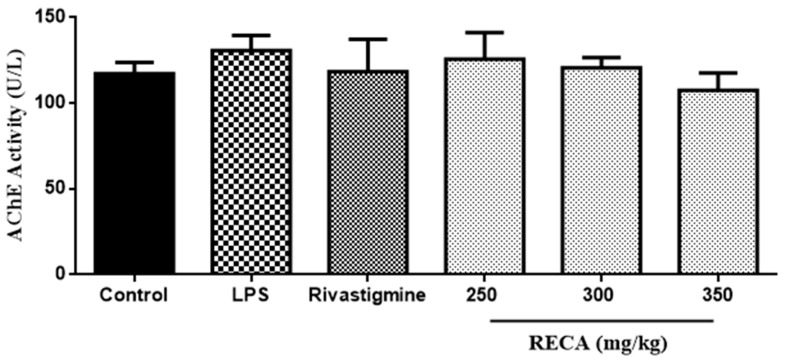
Effect of RECA on acetylcholinesterase activity in vivo. LPS = lipopolysaccharides. Results are expressed as the mean ± SEM in triplicate.

The effect of increasing the concentration of RECA on AChE activity was further evaluated by using the LPS-induced Sprague Dawley rats’ model. Based on Figure 8, the LPS-induced group showed an elevation in AChE activity as compared to control. Treatment of rats with RECA after the LPS injection inhibited LPS-induced enhancement in AChE activity, in a dose-dependent manner. Moreover, it also found that the AChE suppressing effect of the RECA-treated group at a median dosage (300 mg/mL) was comparable to the reference drug group, rivastigmine.

### 2.7. Inhibitory Effect of RECA on Anti-Inflammatory Activities In Vivo

**Figure 9:**
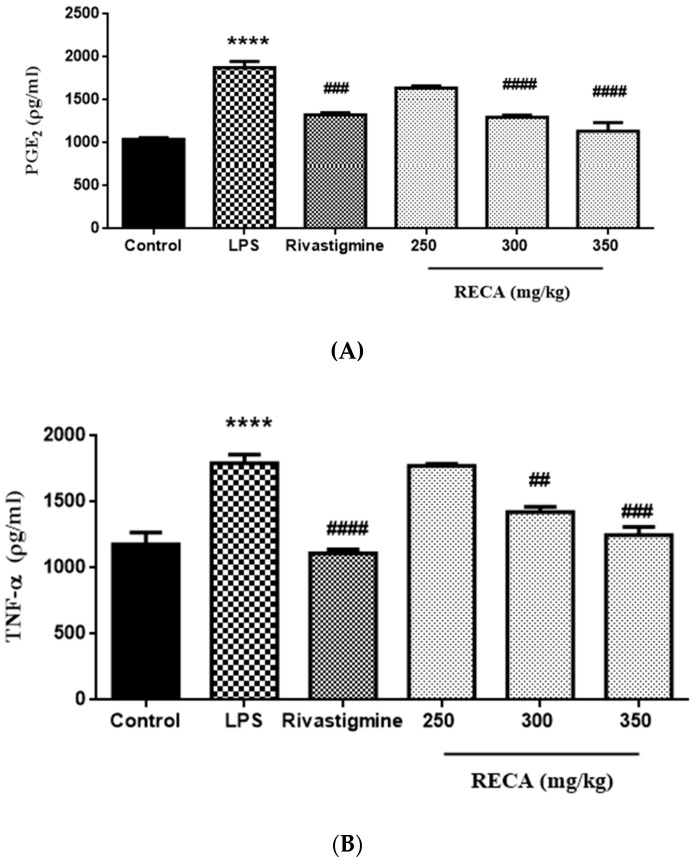
Effect of RECA on (**A**) PGE_2_ and (**B**) TNF-α expressions. Results are expressed as the mean ± SEM in triplicate. LPS = lipopolysaccharides. *** *p* ≤ 0.001 and **** *p* ≤ 0.0001 compared to control group, whereas, ^#^ *p* ≤ 0.05, ^##^ *p* ≤ 0.01, ^###^ *p* ≤ 0.001, and ^####^ *p* ≤ 0.0001 compared to LPS-induced group.

The effects of RECA on the PGE_2_ and TNF-α levels are shown in Figure 9A,B. Rats induced with LPS alone exhibited a significant (**** *p* ≤ 0.0001) elevation in both levels of PGE_2_ and TNF-α, as compared to the respective control group. The rivastigmine group, on the other hand, significantly (^###^ *p* ≤ 0.001 and ^####^ *p* ≤ 0.0001) suppressed the PGE_2_ and TNF-α levels. Significant reductions (^##^ *p* ≤ 0.01, ^###^ *p* ≤0.001 and ^####^ *p* ≤ 0.0001) in both mediators’ levels were also observed in RECA-treated groups at doses of 300 and 350 mg/kg; in contrast, the RECA-treated group at a dose of 250 mg/kg had no significant effect on the level of both mediators.

### 2.8. Inhibitory Effect of RECA on Antioxidant Activity In Vivo

**Figure 10:**
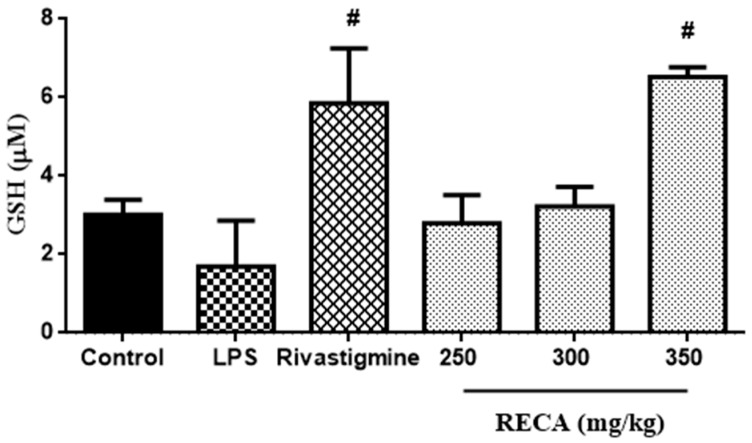
Effect of RECA on GSH level. LPS = lipopolysaccharides. Results are expressed as the mean ± SEM in triplicate. ^#^ *p* ≤ 0.05 compared to LPS-induced group.

In order to elucidate the anti-oxidant activity of RECA in the LPS-induced Sprague Dawley rats’ model, the reduced glutathione (GSH) level was quantified. As shown in Figure 10, the GSH level was depleted in the LPS-induced group. In contrast, the rivastigmine and RECA-treated groups at the highest dosage (350 mg/kg) significantly (^#^ *p* ≤ 0.05) elevated the level of GSH as compared to the group that induced with LPS alone. The effect of the RECA-treated group at a dosage of 350 mg/kg was more pronounced than the standard drug rivastigmine.

## 3. Discussion

The present study reports the capability of ethanolic extract of *C. asiatica* (RECA) in inhibiting acetylcholinesterase (AChE) activity and its possible association in suppressing the elevated expression of pro-inflammatory cytokine/mediators and oxidative stress via in vitro and in vivo models of Alzheimer’s disease (AD).

Even though AD is manifested by the accumulation of neurofibrillary tangles and amyloid plaques, several pieces of evidence suggest the involvement of AChE in the pathogenesis of AD [3,32,33]. Severe loss of cholinergic neurons in brain potentially gives a great impact in all aspects of cognition and behavior in AD patients; hence, drugs targeting the cholinergic system represent the most popular option so far to treat AD patients [34,35]. The human neuroblastoma SH-SY5Y cell line is known to be a relevant cellular model for biochemical investigations on AD, with respect to molecular and neurochemical parameters [36,37]. However, this cell line is rapidly dividing and expresses immature neuronal markers by displaying low morphological and biochemical features of mature neurons [38]. Therefore, as a representative cell model to evaluate the inhibitory effect on AChE activity in vitro, differentiation of SH-SY5Y cells by the non-toxic and supraphysiologic concentration of all-*trans*-retinoic acid (ATRA) was used in this study.

**Figure 11:**
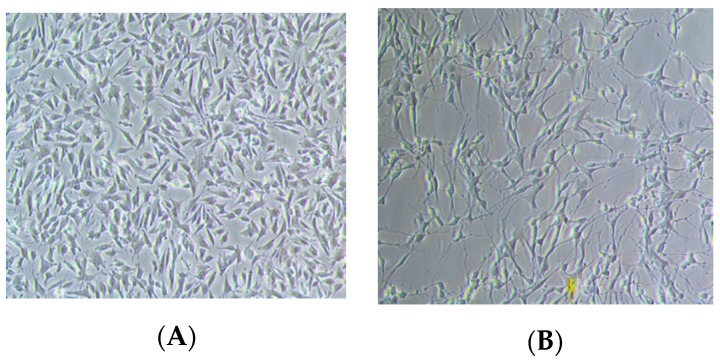
Phase contrast microscopy of neuroblastoma cells, SH-SY5Y undergoing in vitro neural differentiation. (**A**) Undifferentiated cells and (**B**) differentiated cells at 20× magnification.

In order to produce more neurons with a cholinergic phenotype and maximize basal AChE activity, SH-SY5Y cells are commonly induced to differentiate by the addition of retinoic acid (RA) [39,40]. RA has been shown to induce cellular differentiation through various mechanisms, in such a halting cell cycle progression out of G0/G1, enhancing expression of the cyclin-dependent kinase (CDK) inhibitors p21 and p27^Kip1^ and the anti-apoptotic proteins Bcl-2 and Bcl-xL, and increasing PI3K/AKT activity, which plays a role in neurite development and differentiation [41]. To address whether RA-differentiated SH-SY5Y cells are functionally mature in this present study, morphological changes of the cells were observed as in Figure 11, in which the differentiated cells manifested themselves with long and extensively branched neurites that connect to surrounding cells, as compared to undifferentiated cells [42]. The present study shows that treatment of differentiated cells with RECA for 24 h had significantly suppressed the elevated AChE activity with IC_50_ values of 57.47 ± 13.55 μg/mL.

RECA’s inhibition of the AChE enzyme could boost the amount of neurotransmitter acetylcholine and thus enhance synaptic transmission in the AD brain. It is well known that the most significant modifications in AD patients are decreased levels of acetylcholine (ACh) in the brain’s cortex and hippocampus. Consequently, inhibition of AChE, in which the enzyme responsible for ACh hydrolysis in the cholinergic synapse interaction, may be a suitable target for AD therapy [43]. Results from Orhan et al. [44] demonstrated that ethanolic *C. asiatica* extract with 10.78% content of asiaticoside and madecassoside displayed close to 50% inhibition against AChE at 200 μg/mL. Therefore, the AChE inhibition observed in RECA could be attributed by the high proportion of triterpene saponins such as asiaticoside and madecassoside presented in the extract, as these bioactive components were reported to be responsible for its several beneficial pharmacological activities [45].

Neuroinflammation has been implicated to play a pivotal role in facilitating the development of several events in the pathological cascade of AD [46,47]. Microglia cells are the resident immune cells of the central nervous system (CNS) networks and can specifically interact with neurons in the brain once activated [48]. However, uncontrolled activation of microglia by pathological triggers, like neuronal death or protein aggregates, could result in the excessive release of various pro-inflammatory cytokines and neurotoxic agents. These will further lead to CNS damage and disease pathology [49]. On this basis, intervention against the activation process of microglia therefore presents a possible critical target in AD therapy.

In this study, the anti-inflammatory and antioxidant effects of RECA were evaluated against LPS-stimulated RAW 264.7 microglia cells. It is known that for the exposure of microglia cells to inflammatory stimuli, as in this study, LPS was preferred. It exacerbates a wide array of inflammatory mediators including pro-inflammatory cytokines, such as NO, PGE_2_, and TNF-α, which are deleterious to neurons and oligodendrocytes [50]. However, treatment of the activated cells with RECA significantly suppressed the elevated pro-inflammatory cytokine/mediators in a concentration-dependent manner, with the highest concentration (1000 µg/mL) showing remarkable inhibition in all assays. Among all, RECA displayed strong inhibition against nitrite and these inhibitory effects on inflammatory activities observed in RECA were not attributable to its cytotoxic effect, as assessed by MTT. This trend of inhibition clearly confirmed that madecassic acid plays an important role in the anti-inflammatory activity of *C. asiatica* as compared to their glycosides. Therefore, in the present study, RECA reversed the inflammatory activities moderately may be due to the smaller amount of madecassic acid presents in the extract.

Perpetual overactivation of microglia causes an overwhelming production of ROS that promotes oxidative stress and downregulates the level of antioxidants, such as reduced glutathione (GSH) [51]. GSH is the most abundant intracellular non-protein thiol in cells and plays a central role in maintaining cellular redox status. By increasing the GSH content, it would be expected to reduce the ROS level and antagonize apoptotic signals [52]. In support of previous studies, LPS exposure was shown to induce oxidative stress via accelerating the ROS level and, at the same time, GSH depletion [53,54,55]. However, treatment with RECA in the present study suppressed the oxidative changes stimulated by LPS. These inhibitory effects were associated with the reduction of ROS level and stimulation of GSH content, indicating the role of RECA in oxidative balance. A study done by Sasmita et al. [56] indicated that cells treated with the madecassoside at a maximum non-toxic dose (MNTD) were able to suppress the intracellular ROS generations by downregulating the gene and protein expression of pro-neuroinflammatory components. Therefore, the antioxidative properties observed in RECA could be attributed to the high proportion of madecassoside in the extract.

Generally, treatment with RECA in LPS-stimulated macrophages enhanced the GSH content in a concentration-dependent manner up to 125 µg/mL and GSH content at this concentration was observed to be better than the reference drug, α-tocopherol. In contrast, incubation of activated microglia cells with RECA at a further concentration from 250 µg/mL to 1000 µg/mL elicited suppression effect on the GSH content. Interestingly, this trend was found almost similar with the study done by Naidoo et al. [57], in which *C. asiatica* was observed to enhance the GSH content up to 0.2 mg/mL before it started to decrease at further concentrations. To the best of our knowledge, no previous literature has reported the effect of *C. asiatica* in attenuating GSH content in activated RAW 264.7 cells; hence, these findings warrant further investigation.

In order to ascertain whether the in vivo use of RECA can offer the same anti-acetylcholinesterase, anti-inflammatory, and antioxidative effects, we further examined the effect of RECA on the Sprague Dawley rats subjected to LPS injection. The establishment of an appropriate animal model for researching neuroinflammation associated with neurodegeneration in neurodegenerative diseases is very crucial. A large body of experimental evidence has demonstrated that LPS is one of the most suitable inducers for studying the mechanism of neuroinflammation and oxidative stress in animal models, which closely resembles AD [58,59,60]. A single injection of LPS intraperitonially caused neuroinflammation in rats via the TLR-4 signaling pathway, which further activates various intracellular molecules that affect the expression of a plethora of steady-state transcripts such as TNF-α, NO, and PGE_2_ in the serum and brain homogenates [59,61]. In addition, the up-regulated of AChE activity upon the LPS injection in the present study was found in consonance with preceding reports [62,63,64]. This observation might reflect the deficit of cholinergic neurotransmission in the brain and further supports the impression that altered activity of AChE that has been found in patients with AD is attributed by the overexpression of pro- and anti-inflammatory cytokines [65]. Conversely, post-administration of RECA attenuated the AChE activity in a dose-dependent manner where the highest dosage showed a comparable AChE inhibition as rivastigmine. This finding was found in line with our previous work in which RECA showed a protective effect against LPS-pretreated rats [66]. Therefore, it is worth noting that RECA possesses both curative and protective properties as an AChE inhibitor.

In the current study, the depletion of GSH content in LPS-induced rats indicates that there was an overexpression of free radical generation and that the reduced GSH became depleted as it confers protection against oxidative stressors. According to an autopsy study, a magnitude of GSH depletion was found in the AD cingulated cortex and AD substantia innominate, and these unfavorable events were correlated with a decline in cognitive functions [67,68]. Nonetheless, post-administration of RECA has demonstrated a significant increment in GSH content and a reduction of TNF-a and PGE_2_ levels in LPS-induced rats. Besides, it is also observed that GSH content at the highest dosage of RECA (350 mg/kg) was more prominent as compared to rivastigmine. Research has shown that intranasal GSH administration in patients with Parkinson’s disease (PD) elevates GSH levels in the brain [69], whereas clinical studies involving GSH have shown that the ingested GSH has important nutraceutical benefits for human health to improve oxidative stress and human protection [70,71,72]. In addition, the study of individuals with mild cognitive impairment (MCI) showed that the rate of GSH significantly elevated in both the anterior and posterior cingulate regions in patients with MCI as compared to the healthy control participants. The increase in GSH level was explained as an early compensatory or neuroprotective response [73]. Such findings offer important insight into the importance of an effective brain antioxidant system during longevity [74]. Indeed, we postulate that RECA may emerge its antioxidant property by reinforcing the GSH content, which not only serves as an antioxidant agent but also plays as a protective role against inflammatory pathologies in the inflamed brain.

Through the comprehensive analysis of the data referred to above, we can presume that RECA has the capability in quashing the elevated level of AChE, inflammation, and oxidative stress activities.

## 4. Materials and Methods

### 4.1. Chemicals and Reagents

All solvents were HPLC grade, purchased from RCI Labscan (Bangkok, Thailand). The chemical standards of asiaticoside, madecassoside, asiatic acid, and madecassic acid, were purchased from Chemfaces (Wuhan, Hubei, China). Dulbecco’s modified Eagle’s medium (DMEM), Eagle’s minimum essential medium (MEM), fetal bovine serum (FBS), trypsin (0.25%, 1 mM EDTA), and penicillin streptomycin were obtained from Nacalai Tesque (Kyoto, Japan). Ham’s F-12K (Kaighn’s) Medium was obtained from Gibco, Thermo Fisher Scientific, Inc. (Waltham, MA, USA). Dimethyl sulfoxide, LPS from *Escherichia coli* (O26:B6), 3-[4,5-dimethylthiazol-2-yl]-2,5-diphenyltetrazolium bromide (MTT), 2,7-dichlorofluorescein diacetate (DCF-DA), Hanks Balanced Salt Solution, all-*trans*-retinoic acid, and phosphate-buffered saline (PBS) were obtained from Sigma-Aldrich (St. Louis, MO, USA). The triterpenoid standards, asiaticoside, madecassoside, asiatic acid, and madecassic acid were purchased from ChemFaces Natural Products Co., Ltd., China. AChE activity was measured by using QuantiChrom™ Acetylcholinesterase Assay kit (BioAssay Systems, Hayward, CA, USA). The Griess reagent kit for nitrite determination was purchased from Promega™ Griess Reagent System (Promega Biotech Co., Ltd., Madison, WI, USA). The enzyme-linked immunosorbent assay (ELISA) kit for TNF-α measurement was obtained from ElabScience (Rockford, IL, USA), and PGE_2_ and GSH measurements were obtained from Cayman Chemical Company (Ann Arbor, MI, USA). All of the other reagents were of the highest commercially available grade.

### 4.2. Plant Material Preparation and Extraction

Raw materials were obtained from Herbagus Trading, Pulau Pinang, Malaysia. The whole plant was washed, cleaned, and oven-dried at 40 °C. The powdered plant material was extracted using a standard extraction protocol at the extraction facility of the Institute of Bioproducts Development (IBD), Universiti Teknologi Malaysia. The extraction was carried out by using 95% denaturated ethanol for 8 h at a temperature of 60 °C. The yield of crude extract with regard to the weight of dried plant material was about 16%. This extract was designated as raw-extract *C. asiatica* (RECA). Voucher specimen (CA-K017) was prepared and deposited in the Atta-ur-Rahman Institute for Natural Product Discovery, UiTM Puncak Alam (Selangor, Malaysia) for future reference.

### 4.3. Fingerprint Chromatogram Assessment

Chromatographic profiling of RECA and its standards was performed by using a Thermo Scientific™ UltiMate™ 3000 HPLC system coupled to a diode array detector (detector wavelength 200 nm), equipped with an ultra-high pressure pump and vial sampler, as described by previous literature with slight modifications [75]. Chromeleon chromatographic software was used for data collection and processing. Chromatographic separation was carried out on a reversed-phase Ascentis^®^ C18 HPLC column (Supelco, USA) (15 cm × 4.6 mm i.d., 5 μm) with injection volume of 100 μL and a flow rate of 1.0 mL/min. The acquisition time for the analysis was 55 min using a mobile phase with ultra-pure water and HPLC grade acetonitrile, as shown in Table 2A,B, respectively. The concentration of sample for each analysis was 10 mg/mL dissolved in methanol-water (7:3) and filtered through a 0.22 µM nylon membrane syringe filter, prior to the injection. Standard solutions for the evaluation of linearity were prepared over a concentration range of 10, 50, 100, 500, and 1000 ppm in methanol:water (1:1). Quantification of marker compounds was done using calibration curves constructed from the standards and the calibration curve criteria were accepted, with r^2^ > 0.998 indicating an acceptable fit. The relative amount of the compound was expressed as milligram per gram of extract.

### 4.4. Cell Culture

Human neuroblastoma cells, SH-SY5Y, and murine macrophage RAW 264.7 were obtained from the American Type Culture Collection (ATCC no. CRL-2266 and no. TIB-71™, Manassas, VA, USA). SH-SY5Y was cultured in the mixture of minimum essential medium (MEM) Eagle with Earle’s salt and F12-K medium in a 1:1 ratio, whereas RAW 264.7 was cultured in Dulbecco’s modified Eagle medium (DMEM). Both were supplemented with 10% heat-inactivated fetal bovine serum (FBS) and 1% penicillin/streptomycin in a humidified incubator maintained at 37 °C under 5% CO_2_/95% air. The media were changed every 2 days and subculture was performed when cells reached 80% confluence. Cell numbers were assessed using a hemocytometer. For cell viability assay, SH-SY5Y and RAW 264.7 cells were plated at density 10 × 10^3^ cells/well and 30 × 10^3^ cells/well in 96-well plates. Meanwhile, for assay to determine inhibition of AChE activity, SH-SY5Y cells were plated at a density of 5 × 10^5^ cells/well in 6-well plates. For assays to determine inhibition activity of NO, TNF-α, PGE_2_, and ROS productions, RAW 264.7 cells were plated at density 30 × 10^3^ cells/well in 96-well plates; whereas, to determine the production of GSH level, RAW 264.7 cells were plated at density 8 × 10^5^ cells/well in 6-well plates.

### 4.5. Determination of Cell Viability Assay

The cytotoxic effects of RECA on the SH-SY5Y and RAW 264.7 cell lines were determined colorimetrically using the MTT method, which is based on the reduction of tetrazolium salt by mitochondrial dehydrogenase in viable cells. Serial two-fold dilutions of the extracts were added to the wells in triplicate with an increasing concentration (3.91–1000 µg/mL) and incubated for 24 h. A solution of the MTT reagent, 3-(4,5-dimetylthiazol-2-yl)- 2,5-diphenyl tetrazolium bromide (5 mg/mL PBS) was added to both cells in each well and incubated for the next 3 h. Resulting formazan was solubilized with 100 μL dimethyl sulfoxide (DMSO). The plate was shaken for 15 min and absorbance determined at 540 nm using a SPECTROstar Nano plate reader (BMG Labtech Inc., Cary, NC, USA). Values were calculated in comparison to the control cells.

### 4.6. Determination of Anti-Acetylcholinesterase Activity

The AChE inhibitory activity was measured using Ellman’s method, as reported previously [66]. SH-SY5Y cells were induced to differentiate into neuronal cells by all-*trans*-retinoic acid (ATRA; 10 µM). After differentiation, culture media was removed, and cells incubated with various concentrations of RECA (3.91–1000 µg/mL) for the next 24 h. Eserine was used as the reference drug. Cell lysates were assayed into 96-well microplates for acetylcholinesterase activity as per manufacturer’s instructions. The intensity of the color change was measured at 412 nm of the 2nd and 10th min using the microplate reader.

### 4.7. Determination of Anti-Inflammatory Activities

#### 4.7.1. Nitrite Production

Briefly, RAW 264.7 cells were plated and left incubated for 24 h. Dexamethasone was used as the standard drug. Cells were induced with LPS for 24 h prior to the treatment with RECA/dexamethasone at the indicated concentration for the next 24 h. The concentration of nitrite in the cultured media was measured at 540 nm. Nitrite was quantified by an external standard, using sodium nitrite to generate a standard curve.

#### 4.7.2. Prostaglandin E2 (PGE2) and Tumor Necrosis Factor Alpha (TNF-α) Levels

In brief, RAW 264.7 cells were stimulated with LPS (4 µg/mL) 24 h prior to the treatment with several concentrations of RECA (3.91–1000 µg/mL) for the next 24 h. The PGE_2_ and TNF-α productions were quantified by measuring its cultured media at 412 nm.

### 4.8. Determination of Antioxidant Activities

#### 4.8.1. Intracellular Reactive Oxygen Species (ROS) Production

Assay was performed as described in Section 4.7.1 with α-tocopherol was used as the standard drug. At the end of treatment, the medium was discarded, and the wells were gently washed thrice with Hanks Balanced Salt Solution (HBSS). Cells were stained with 20 µM H_2_DCF-DA and left incubated for 45 min at 37 °C in the dark. Then, cells were gently washed thrice with HBSS to remove excess dye solution. Finally, an additional 200 µL of HBSS was loaded to the cells and the fluorescence was detected in a spectrophotometer at an excitation wavelength of 485 nm and an emission wavelength of 520 nm.

#### 4.8.2. Intracellular Reduced Glutathione (GSH) Level

In brief, RAW 264.7 cells were stimulated with LPS (4 µg/mL) 24 h prior to the treatment with several concentrations of RECA (3.91–1000 µg/mL) for the next 24 h. α-tocopherol was used as the standard drug. The GSH level was quantified by measuring the cell lysates at 450 nm as per manufacturer’s instructions.

### 4.9. Animal Study Design

**Figure 12:**
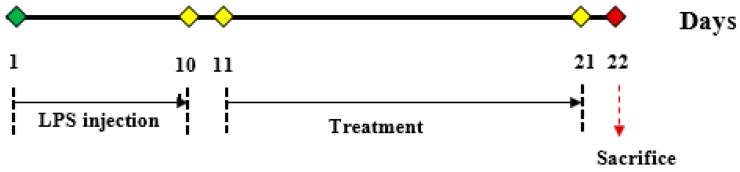
Experimental study design.

Adult male Sprague Dawley rats, weighing approximately 200–250 g, were procured from the Laboratory Animal Facility and Management Faculty of Pharmacy (LAFAM), UiTM Puncak Alam, Selangor. Rats were housed in individually ventilated cages (IVC) cage and maintained under standard laboratory conditions that were automatically kept at 21–25 °C and relative humidity at 45%–65% with a controlled light–dark cycle. All rats had free access to standard laboratory food and tap water ad libitum. Rats were housed in groups and acclimatized for at least 7 days before using them for experiments. Experiments were carried out between 0800 h to 1800 h. The protocol was approved by the Committee on Animal Research and Ethics of the Universiti Teknologi MARA (UiTM) (Reference No: 600-FF (PS. 17/2/1); dated 28 August 2017). The laboratory animals were handled and managed in accordance to the Guide for the Care and Use of Laboratory Animal (National Research Council 1996). Thirty-six rats were randomly assigned into 6 groups (6 rats/group; Table 3) for biochemical analysis. Except for untreated control, all groups were injected intra-peritoneally (i.p) with LPS at a concentration of 250 µg/kg for first the 10 days, and subsequently from day 11 to day 21, RECA (250, 300 and 350 mg/kg) and rivastigmine (5 mg/kg) were administered through oral gavage. The experimental study design is shown in Figure 12. All the drugs (RECA, rivastigmine, and LPS) were freshly prepared before administration. The rats were sacrificed by cervical decapitation under light anesthesia for biochemical assessment on day 22.

The doses of RECA, rivastigmine, and LPS administered were selected based on previous work [66].

#### 4.9.1. Vehicles

LPS was diluted in phosphate buffer saline (PBS). Rivastigmine (5 mg/kg) and RECA (250, 300, and 350 mg/kg) were suspended in distilled water for oral administration.

#### 4.9.2. Biochemical Analyses

Immediately after decapitation, the whole brain was carefully removed from the skull, washed, and homogenized in ice-cold PBS to prepare 10% (*w*/*v*) homogenate by using a handheld rotor-stator homogenizer (TissueRuptor, QIAGEN, Germany). The brain homogenate was centrifuged at 3000 rpm at 4 °C for 15 min to remove cellular debris and resultant cloudy supernatant was collected and used for biochemical assessment such as quantification of AChE, anti-inflammatory, and antioxidant activities as per the manufacturer’s instructions.

#### 4.9.3. Statistical Analysis

For statistical analysis, each of the experimental values was compared with its corresponding control. Results were expressed as the mean ± standard error of the mean (SEM). Mean differences among groups were evaluated by one-way analysis of variance (ANOVA) using the GraphPad Prism 6.0 software (SAS Institute, NC, USA). Post-hoc comparisons between groups were made using Tukey’s test. At least, at the level of *p* < 0.05, the results were considered as statistically significant.

## 5. Conclusions

In conclusion, current findings suggest that the raw-extract of *C. asiatica* (RECA) possesses capability in quashing the elevated level of acetylcholinesterase (AChE), inflammation, and oxidative stress activities in vitro and in vivo. The combination of findings from in vitro and animal studies in the present work are useful for current knowledge contribution and future research needs. Besides, it may also provide important insights that *C. asiatica* or compound derived from it can be considered as potential relevance in managing AD.

## Figures and Tables

**Table 1:** Triterpene concentration of RECA.

Types of Triterpenes	Retention Time (min)	Concentration of Triterpenes in RECA ± SEM (mg/g)	% of Dry Plant
Madecassoside	9.077	231.44 ± 7.05	3.70 ± 0.11
Asiaticoside	11.393	223.21 ± 5.63	3.57 ± 0.09
Madecassic acid	22.207	118.95 ± 8.47	1.91 ± 0.14
Asiatic acid	25.417	108.37 ± 2.25	1.73 ± 0.04

SEM = standard error of the mean.

**Table 2:** Gradient condition for HPLC.

Time (min)	Pump A, Water (%)	Pump B, Acetonitrile (%)
0	80	20
15	65	35
30	35	65
35	20	80
40	20	80
45	80	20
55	80	20

**Table 3:** Type of groups.

Animal Group	Treatment
(i)	Saline (ip) + distilled water
(ii)	RECA (250 mg/kg, PO) + LPS (250 µg/kg, ip)
(iii)	RECA (300 mg/kg, PO) + LPS (250 µg/kg, ip)
(iv)	RECA (350 mg/kg, PO) + LPS (250 µg/kg, ip)
(v)	LPS-treated (250 µg/kg, ip)
(vi)	Rivastigmine (5 mg/kg, PO) + LPS (250 µg/kg, ip)
